# High-performance varistors simply by hot-dipping zinc oxide thin films in Pr_6_O_11_: Influence of temperature

**DOI:** 10.1038/srep41994

**Published:** 2017-02-03

**Authors:** Yang Wang, Zhijian Peng, Qi Wang, Chengbiao Wang, Xiuli Fu

**Affiliations:** 1 School of Engineering and Technology, China University of Geosciences, Beijing 100083, PR China; 2 State Key Laboratory of Information Photonics and Optical Communications, and School of Science, Beijing University of Posts and Telecommunications, Beijing 100876, P. R. China

## Abstract

High-performance ZnO-Pr_6_O_11_ thin-film varistors were fabricated simply by hot-dipping oxygen-deficient zinc oxide thin films in Pr_6_O_11_ powder. The films had a composition of ZnO_0.81_ and a thickness of about 200 nm, which were deposited by radio frequency magnetron sputtering a sintered zinc oxide ceramic target. Special attention was paid on the temperature dependence of the varistors. In 50 min with hot-dipping temperature increased from 300–700 °C, the nonlinear coefficient (*α*) of the varistors increased, but with higher temperature it decreased again. Correspondingly, the leakage current (*I*_L_) decreased first and then increased, owing mainly to the formation and destroying of complete zinc oxide/Pr_6_O_11_ grain boundaries. The breakdown field (*E*_1mA_) decreased monotonously from 0.02217 to 0.01623 V/nm with increasing temperature (300–800 °C), due to the decreased number of effective grain boundaries in the varistors. The varistors prepared at 700 °C exhibited the optimum nonlinear properties with the highest *α* = 39.29, lowest *I*_L_ = 0.02736 mA/cm^2^, and *E*_1mA_ = 0.01757 V/nm. And after charge-discharge at room temperature for 1000 times, heating at 100 or 250 °C for up to 100 h, or applying at up to 250 °C, the varistors still performed well. Such nanoscaled thin-film varistors will be very promising in electrical/electronic devices working at low voltage.

Nowadays technological development aims for designing and manufacturing various kinds of super-large-scale integrated circuits, which require increasing quantity of miniaturized devices including various varistors working at low voltage. Up to now, most of the commercially applied varistors have been ZnO-Bi_2_O_3_ based electronic ceramic devices, which are widely used in various electrical circuits, electronic devices and electrical power systems to counteract against dangerous over-voltage surges, due to their highly nonlinear current-voltage (*I-V*) characteristics and outstanding energy handling capabilities^1^,^2^.

For conventional ZnO-Bi_2_O_3_ based ceramic varistors, they are prepared by sintering ZnO powder with a small amount of the so-called varistor forming oxide (VFO) Bi_2_O_3_, which is essential for imparting the nonlinearity of ZnO ceramics, while other oxides, such as nickel oxide, manganese oxides, rare metals oxides and so on, are also added in small amount to further enhance the non-linearity of the varistors^3^,^4^. It is well-known that their nonlinear *I-V* characteristics are controlled by the well-recognized double Schottky barrier mechanism, which is triggered by the contacting layer between ZnO grains (the core) and the VFO Bi_2_O_3_ thin films (the shell) on them formed during sintering^5^. Thus, to meet the requirement of miniaturized low-voltage devices, complicated and relatively costly processes such as tape casting, higher temperature sintering and micro machining/manufacturing have to be developed, attempting to promote the growth of ZnO grains and/or reduce the thickness of the electronic components. But owing to the complicated processes and low product consistence during preparing low-voltage ZnO chip varistors by ceramic methods, the alternative films deposition methods aiming at reducing the thickness of the components are more desirable^6^. To date, several physical and chemical methods have been proposed to prepare ZnO film varistors, such as magnetron sputtering, molecular beam epitaxy, pulsed laser deposition, chemical vapor deposition, spray pyrolysis, sol-gel process, and so on^7^,^8^,^9^,^10^. Among them, magnetron sputtering is preferable due to its relatively high deposition rate, low substrate temperature, high film thickness uniformity, good controllability and high repeatability^10^. And many composite ZnO-Bi_2_O_3_ and multilayered ZnO/Bi_2_O_3_ thin-film varistors were reported^7^,^8^. But to prepare such varistors, dual (Zn/ZnO and Bi/Bi_2_O_3_) or various composite ZnO-Bi_2_O_3_ sputtering targets, and even more complicated precursors are needed. And because of their low controllability of material composition and structure, the product consistence of the already reported ZnO-Bi_2_O_3_ based thin-film varistors has been not satisfactory. What’ worse, because magnetron sputtering is characteristic of low-temperature deposition, effective double Schottky barriers at the grain boundaries with well-built contacting layers between the ZnO grains and Bi_2_O_3_ thin films are not easy to form. As a result, the nonlinear coefficients of such ZnO-Bi_2_O_3_ based thin-film varistors are very low (normally with a value lower than 10).

Considering the afore-mentioned facts, to improve the performance of ZnO-Bi_2_O_3_ based thin-film varistors, in our previous work, by radio frequency (RF) magnetron sputtering of a sintered zinc oxide ceramic target, oxygen-deficient zinc oxide thin films with tunable composition and electrical resistivity were deposited^11^, and through hot-dipping the obtained oxygen-deficient zinc oxide thin films in Bi_2_O_3_ powder, highly nonlinear binary ZnO-Bi_2_O_3_ thin-film varistors with an optimum nonlinear coefficient of 15.1 (basically being an equivalent to ZnO-based bulk ceramic varistors) were obtained, because the effective double Schottky barriers were well built up at the grain boundaries^6^. However, such thin-film varistors will still suffer from low high-temperature stability in applications, because at high temperature, the core oxygen-deficient zinc oxide films are easily oxidized and the shell Bi_2_O_3_ thin films are highly volatile.

Moreover, just because Bi_2_O_3_ is used as the VFO, the conventional ZnO-Bi_2_O_3_ based ceramic varistors have a few drawbacks due to the high volatility and reactivity of Bi_2_O_3_ during liquid sintering^12^. The high volatility of Bi_2_O_3_ will change the varistors’ characteristics due to the variation of inter-composition ratio of the additives. The high reactivity of Bi_2_O_3_ will destroy the multi-layer structure of the chip varistors and deteriorate the surge-absorption capabilities due to the decrease of effective grain boundary number^13^. To overcome these problems, ZnO ceramic varistors containing different VFOs have been actively studied, in which ZnO-Pr_6_O_11_ based varistors are most promising in application, due to their advantages over ZnO-Bi_2_O_3_ based varistors in relatively simple two-phase microstructure of ZnO grains and praseodymium oxide intergranular phases. Such structure can reduce the quantity of compositional materials, improve the electrical properties of the ceramics, and keep the composition stable at high-temperature sintering due to the high melting point of Pr_6_O_11_^14^.

Therefore, in this work, we use Pr_6_O_11_ to replace Bi_2_O_3_ in the hot-dipping of oxygen-deficient zinc oxide thin films. Special attention was paid on the temperature dependence of the composition, microstructure and electrical properties of the samples. Surprisingly, ZnO-Pr_6_O_11_ thin-film varistors with a recorded high nonlinear coefficient (39.29) were fabricated. And because of the high-temperature stability of Pr_6_O_11_, the obtained ZnO-Pr_6_O_11_ thin-film varistors presented a very high performance even in elevated temperature applications.

## Elemental composition and chemical state

**Figure 1:**
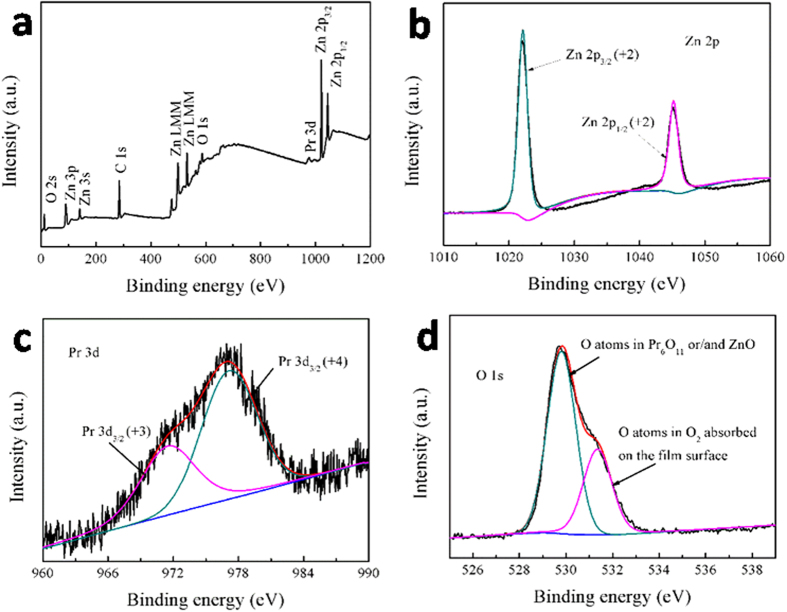
XPS results. (**a**) Full spectrum, and narrow spectra of, (**b**) Zn 2p, (**c**) Pr 3d and (**d**) O 1 s for typical hot-dipped sample (in Pr_6_O_11_ at 700 °C for 50 min in air).

X-ray photoelectron spectroscopy (XPS) analysis indicated that, only Zn 2p and O 1 s peaks could be detected in the applied oxygen deficient zinc oxide thin films, which had a composition of ZnO_0.81_^6^,^11^. After hot-dipping in Pr_6_O_11_ powder at the designed conditions, the elemental composition and chemical state of the obtained samples were also examined by XPS. Typical results on the sample hot-dipped in Pr_6_O_11_ in air at 700 °C for 50 min are presented in Fig. 1. From the full spectrum as shown in Fig. 1a, Zn 2p, Pr 3d and O 1 s peaks could be identified, indicating that some Pr atoms were composite with the zinc oxide film after hot-dipping. Moreover, from Fig. 1b, it can be seen that the Zn 2p narrow spectrum of the hot-dipped samples displays two symmetric peaks. The peaks located at 1022.2 ± 0.1 and 1045.2 ± 0.1 eV can be assigned to Zn 2p_3/2_ and Zn 2p_1/2_ of Zn^2^ oxidation state, respectively^15^. In addition, as seen from Fig. 1c, the asymmetric Pr 3d_3/2_ narrow spectrum can be deconvoluted into two peaks. The strong peak located at 977.1 ± 0.1 eV is corresponding to Pr^4+^ oxidation state, while the weak peak at 971.5 ± 0.1 eV can be assigned to Pr^3+^ oxidation state^16^. And from this spectrum, the percentages of Pr^4+^ and Pr^3+^ oxidation states were further evaluated as 66.7% and 33.3%, respectively. Thus the mean valence of praseodymium atoms in this sample was calculated as 3.67. In other word, the chemical composition of Pr with O is Pr_6_O_11_, which is exactly in agreement with that of the originally applied pure Pr_6_O_11_ powder, implying that Pr_6_O_11_ presents mainly as a separate phase in the sample. Meanwhile, as shown in Fig. 1d, the narrow spectrum of O 1 s of the samples could be fitted into two components. The first component has a binding energy of 529.8 ± 0.1 eV, which can be attributed to the oxygen atoms in ZnO^17^ and Pr_6_O_11_^18^. The second one has a binding energy of 531.4 ± 0.1 eV, which is owing to the adsorbed oxygen (such as O_2_) on the film surface^19^. All these results indicate that the as-presented hot-dipped sample is a ZnO-Pr_6_O_11_ composite. And considering that the originally applied zinc oxide thin films were oxygen deficient with a composition of ZnO_0.81_, such results revealed that the zinc oxide thin films were further oxidized during the hot-dipping.

## Microstructure and elemental distribution

**Figure 2:**
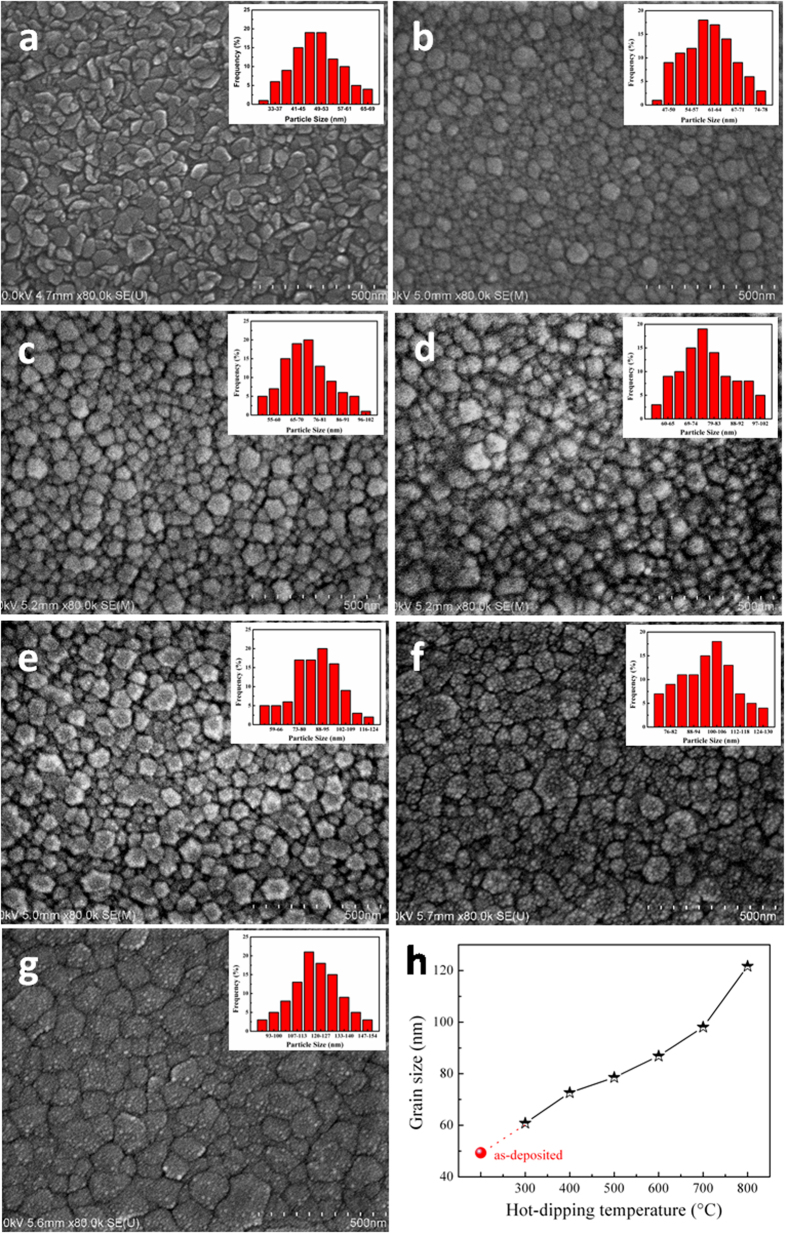
SEM results. FE-SEM surface images of the as-prepared thin films samples hot-dipped in Pr_6_O_11_ for 50 min in air at different temperatures: (**a**) without hot-dipping, (**b**) 300, (**c**) 400, (**d**) 500, (**e**) 600, (**f**) 700 and (**g**) 800 °C. The inset in each image displays the grain size distribution in the films. (**h**) The calculated grain size from the FE-SEM images as a function of the hot-dipping temperature.

Figure 2 presents typical field-emission scanning electron microscopy (FE-SEM) images on the surfaces of the prepared thin films, revealing that the hot-dipping imparts a significant impact on the morphology of the samples. As is seen from Fig. 2a, the surface of the as-deposited ZnO_0.81_ films without hot-dipping is relatively rough with flaky (of obvious edges) and unevenly sized grains. But after the films were hot-dipped, the surface of the films turned out to be smoother and denser, and the grains in the films became more round (see Fig. 2b–g), revealing that the hot-dipping promoted the reforming of the grains in the films, reaching their lower state of energy. Furthermore, with increasing hot-dipping temperature, the mean grain size of the samples increased gradually from 49.37 to 121.68 nm (see the inset of each image and Fig. 2h), indicating that the hot-dipping helped the grain in the films to grow.

**Figure 3:**
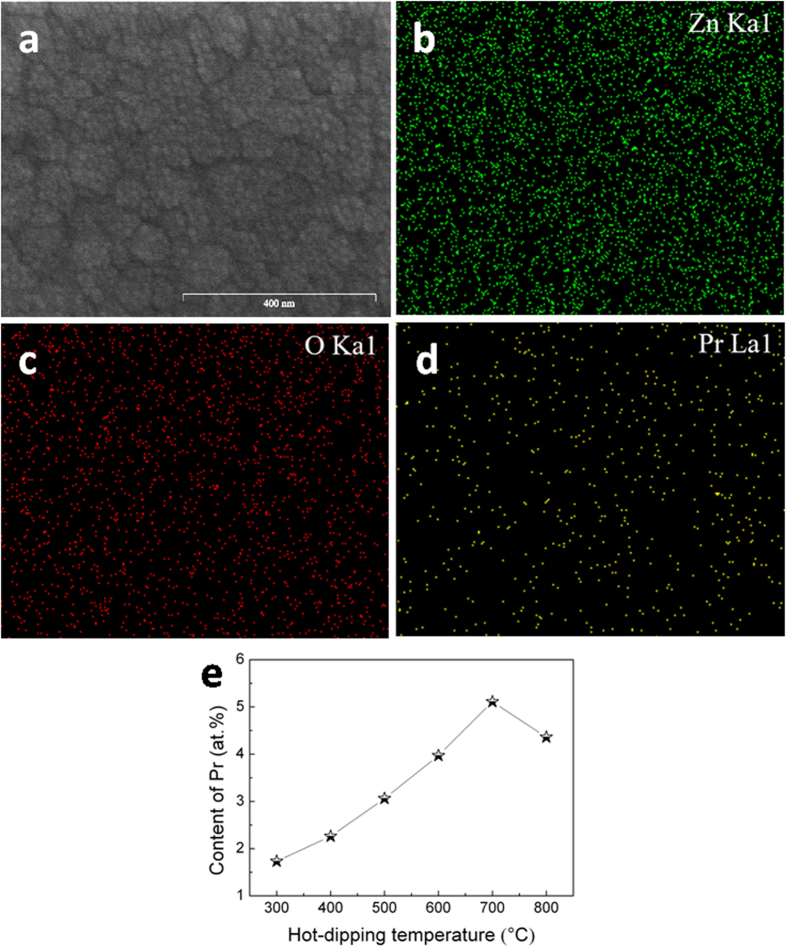
EDX mapping results on the fresh surface of a typical sample prepared by hot-dipping ZnO_0.81_ thin film in Pr_6_O_11_ at 700 °C for 50 min in air. (**a**) EDX scanning area; and (**b–d**) EDX spectra of Zn, O and Pr, respectively. (**e**) The measured content of Pr in all the hot-dipped samples as a function of the hot-dipping temperature.

**Figure 4:**
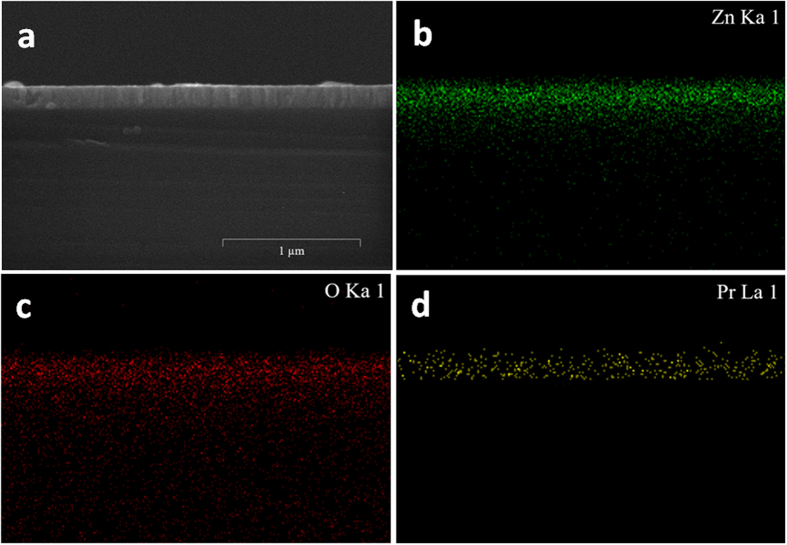
EDX mapping results on the cross-section of a typical sample prepared by hot-dipping ZnO_0.81_ thin film in Pr_6_O_11_ at 700 °C for 50 min in air. (**a**) EDX scanning area; and (**b–d**) EDX spectra of Zn, O and Pr, respectively.

The elemental composition and distribution of all the hot-dipped samples were further examined by energy dispersive X-ray spectroscopy (EDX). Typical results on the fresh surface of the sample prepared by hot-dipping in Pr_6_O_11_ at 700 °C for 50 min in air are shown in Fig. 3a–d. It can be seen that Zn and O atoms are distributed quite uniformly in the samples, but it seems that Pr atoms are scattered mainly at the grain boundary and it is very difficult to estimate if the added Pr_6_O_11_ diffuses into the oxygen-deficient zinc oxide grains during the hot-dipping. This result indicates that the hot-dipping could drive the added Pr_6_O_11_ selectively spread into the grain boundaries of the zinc oxide films, constructing the typical structure of a ZnO-Pr_6_O_11_ varistor. In addition, with increasing hot-dipping temperature, the measured content of Pr in the samples increased initially and decreased somewhat later (see Fig. 3e). When the samples were hot-dipped from 300 to 700 °C, the content of Pr increased from 1.73 to 5.11 at.%. But when the temperature was further increased up to 800 °C, the content of Pr was reduced to 4.36 at.%. For this phenomenon, it can be explained as follows. Because the Pr_6_O_11_ distribution is controlled by thermal diffusion process, so a moderate increase of the hot-dipping temperature is beneficial for its diffusion into the zinc oxide film. When it does not exceed 700 °C, with the increase of hot-dipping temperature, the diffusion speed of Pr_6_O_11_ increases, resulting in increased content of Pr in the samples. However, when it is higher than 700 °C, with further increasing temperature, the content of Pr in the samples decreases, possibly because Pr_6_O_11_ separates out from the samples at enhanced temperature. Moreover, typical results on the cross-section of the sample prepared by hot-dipping in Pr_6_O_11_ at 700 °C for 50 min in air are presented in Fig. 4. It can be seen that the Zn and O atoms are distributed quite homogeneously on the cross-section of the films as they have done on the fresh film surface; and the Pr element also presents a similar non-uniform distribution on the cross-section of the films with that on the film surface. And, Pr has already diffused throughout the whole film without any gradient along the film thickness. The afore-mentioned results indicate that the elemental composition and distribution of the hot-dipped samples are consistent throughout the films.

From the microstructure examination on the film cross-section, it should be noted that all the hot-dipped film samples have a quite homogeneous thickness of about 200 nm, which is almost equal to the original thickness of the as-deposited ZnO_0.81_. This result implies that the hot-dipping would not change the thickness of the samples, which is beneficial to keeping the size of the varistor components.

## Phase composition and defect state

**Figure 5:**
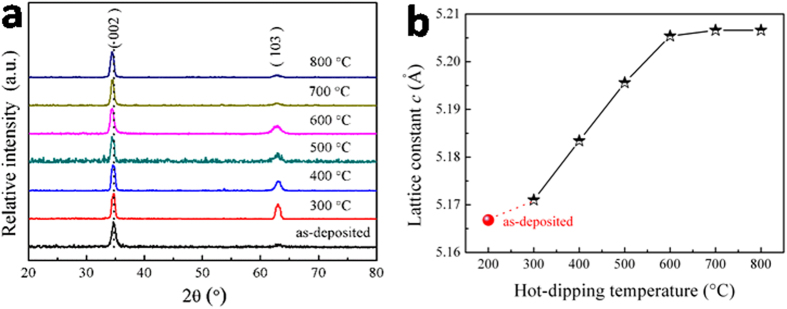
XRD results. (**a**) XRD patterns for the films samples prepared by hot-dipping in Pr_6_O_11_ for 50 min in air at different temperatures. (**b**) Lattice constant *c* calculated from the recorded XRD patterns for the zinc oxide grains as a function of the hot-dipping temperature. For comparison, the results on the as-deposited ZnO_0.81_ film are also presented.

The phase composition and defect state of the film samples were examined by X-ray diffraction (XRD). Figure 5a shows the XRD patterns of the samples prepared by hot-dipping in Pr_6_O_11_ for 50 min in air at different temperatures. To make a comparison, the result on the as-deposited ZnO_0.81_ thin films is also presented. As is seen from this figure, all the samples only display a relatively strong (002) peak and a much weak (103) one, both of which can be indexed to the hexagonal würtzite structure zinc oxide phase (JCPDS card no. 65–3411). In other word, only ZnO phase can be identified without any other extra phases, no matter whether they are hot-dipped or not (also see Extended Data Fig. 1a). This phenomenon is resulted in probably by the fact that, under the applied hot-dipping conditions, the content of Pr_6_O_11_ diffusing into the films is low, which cannot be identified in the XRD detection limit. Considering the high stability of Pr_6_O_11_ (which is stable in air at a temperature of up to 800 °C, as is seen from the Extended Data Fig. 2) and high melting point of Pr_6_O_11_ (2042 °C), the existed praseodymium oxide in the film should be in the form of Pr_6_O_11_ phase. And this result is in accordance with the EDX data as shown in Fig. 3e.

To evaluate the defect state in the samples, the lattice constant *c* of the zinc oxide grains in the prepared samples was calculated from the recorded XRD patterns, and the result is presented in Fig. 5b. For comparison, the result on the as-deposited ZnO_0.81_ film is also presented. The lattice constant of the as-deposited ZnO_0.81_ thin film is 5.1668 Å, smaller than that of stoichiometric ZnO (5.2066 Å)^20^, implying that the applied ZnO_0.81_ thin film is an oxygen-deficient one with lots of oxygen vacancies^11^,^21^. However, after hot-dipping at the designed temperatures, the lattice constant for the samples increased, although all of them were still less than 5.2066 Å (more or less). This result implies that the oxygen-deficient ZnO_0.81_ thin films would be oxidized during hot-dipping because oxygen atoms are easy to diffuse into the films, resulting in zinc oxide films with less oxygen vacancies; but they had not been oxidized completely under the present conditions and thus there were still certain amount of oxygen vacancies in the film samples. In particular, with the hot-dipping temperature increased from 300–600 °C, the lattice constant promptly increased, indicating that the films were being oxidized quickly, resulting in samples with zinc oxide grains of less and less oxygen vacancies. When the hot-dipping temperature increased from 600–800 °C, the lattice constant increased more and more slowly, approaching to 5.2066 Å (the value of stoichiometric ZnO), implying that the samples were gradually oxidized completely with a composition of zinc oxide close to the stoichiometric ZnO. And all these results are similar with the lattice constant change of the as-deposited ZnO_0.81_ film annealed at the same temperature as in hot-dipping (see Extended Data Fig. 1b). Moreover, although the radii of both Pr^3+^ (99 Å) and Pr^4+^ (85 Å) ions are bigger than that of Zn^2+^ ion (74 Å)^22^, the lattice constant of the hot-dipped samples is still smaller that of stoichiometric ZnO, implying that the Pr ions would not diffuse into the zinc oxide grains during the hot-dipping. This result indirectly confirms that Pr_6_O_11_ is mainly distributed at the grain boundary in the process of hot-dipping, which is in agreement with the results recorded by XPS and SEM mapping.

## Varistor properties

**Figure 6:**
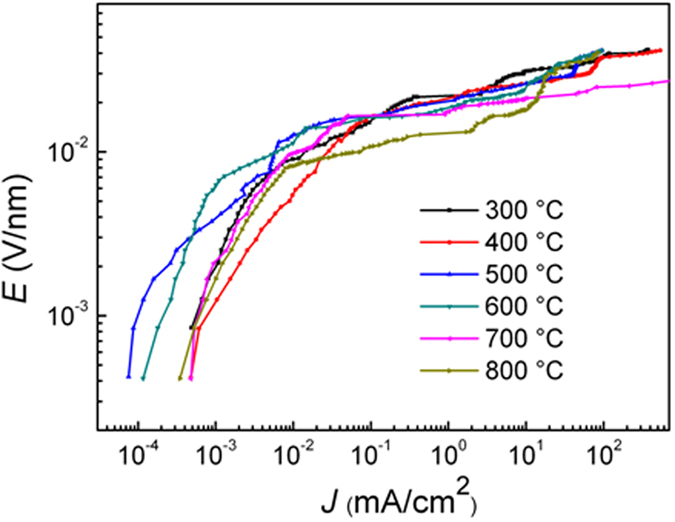
*E-J* characteristic curves of the varistors prepared by hot-dipping ZnO_0.81_ thin films in Pr_6_O_11_ for 50 min in air at different temperatures.

Figure 6 presents the electric field vs current density (*E-J*) characteristic curves of the zinc oxide film varistors prepared by hot-dipping ZnO_0.81_ thin films in Pr_6_O_11_ for 50 min in air at different temperatures. It is well-known that the as-deposited oxygen-deficient ZnO_0.81_ film will display an Ohmic behavior, because there is no double Schottky barrier between their grain boundaries^6^. But after hot-dipping in Pr_6_O_11_ under the designed conditions, all the samples exhibit excellent nonlinear characteristics, indicating they are promising varistors. This result also implies that through hot-dipping in Pr_6_O_11_, an intergranular Pr_6_O_11_-rich phase, which separates the semiconducting ZnO grains, has been well formed, because the presence of electrically active grain boundaries can produce the double Schottky barriers, inducing the non-linear characteristics of the varistor samples^23^.

**Figure 7:**
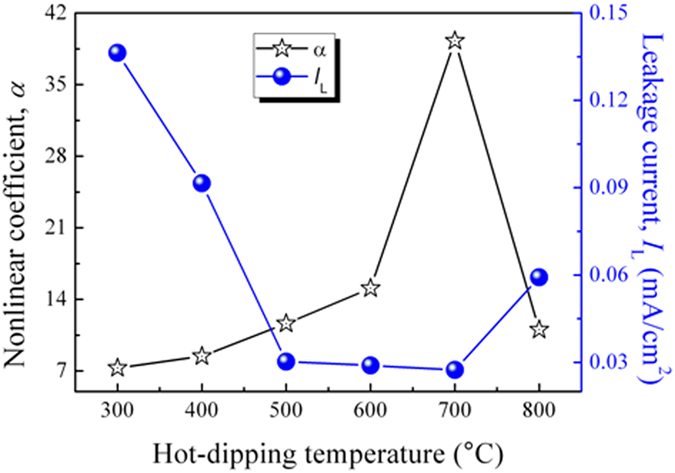
Nonlinear coefficient and leakage current of the zinc oxide film varistors as a function of hot-dipping temperature.

From Fig. 6, the basic electrical parameters of the obtained varistors were calculated, in which nonlinear coefficient (*α*) is the most important one. Figure 7 illustrates the corresponding nonlinear coefficient of the varistors as a function of the hot-dipping temperature (black line marked with stars). As is seen from this figure, with the increase of hot-dipping temperature, the nonlinear coefficient initially increased and then decreased, reaching a maximum of 39.29 (much higher than those of the ZnO-based bulk ceramic varistors^24^) when the varistor was prepared by hot-dipping ZnO_0.81_ thin film in Pr_6_O_11_ at 700 °C for 50 min in air. With increasing hot-dipping temperature when it was less than 700 °C, the nonlinear coefficient of the prepared varistors increased gradually. This is because in the process of hot-dipping, Pr_6_O_11_ would gradually permeate into the zinc oxide grain boundaries in the films, forming an insulating layer at the grain boundaries, finally triggering the nonlinear behavior of a varistor. And because the formation of the intergranular insulating Pr_6_O_11_ layer is determined by thermal diffusion process, as discussed in Section 3.2, a moderate increase of the hot-dipping temperature is beneficial for the diffusion of Pr_6_O_11_ into the zinc oxide grain boundaries in the films. As is seen in Fig. 3e, the content of Pr in the films continuously increases till the hot-dipping temperature increases up to 700 °C. Therefore, with increasing hot-dipping temperature up to 700 °C, the structure of the intergranular insulating layer will become more and more completed. As a result, the nonlinear coefficient of the samples increased. However, when the hot-dipping temperature was higher than 700 °C, the nonlinear coefficient of the samples would gradually decrease with further increasing temperature. This is because Pr_6_O_11_ separates out from the intergranular insulating layer of the zinc oxide film varistors at enhanced temperatures with decreasing content of Pr in the films, destroying the completeness of the intergranular layer, reducing the intergranular insulation resistance, finally leading to the decrease of the nonlinear coefficient of the samples.

Correspondingly, with the increase in the hot-dipping temperature, the leakage current (*I*_L_) initially decreased and then increased, achieving a minimum of 0.02736 mA/cm^2^ for the varistors prepared by hot-dipping ZnO_0.81_ thin film in Pr_6_O_11_ at 700 °C for 50 min in air (see the blue line marked with balls in Fig. 7). With the increase of hot-dipping temperature, the leakage current of the samples shows a trend against the nonlinear coefficient. The high nonlinear coefficient for a varistor would lead to low leakage current because of the formation of a relatively high tunnel current, while the low nonlinear coefficient would result in high leakage current due to the formation of higher thermal excitation radio current^25^.

**Figure 8:**
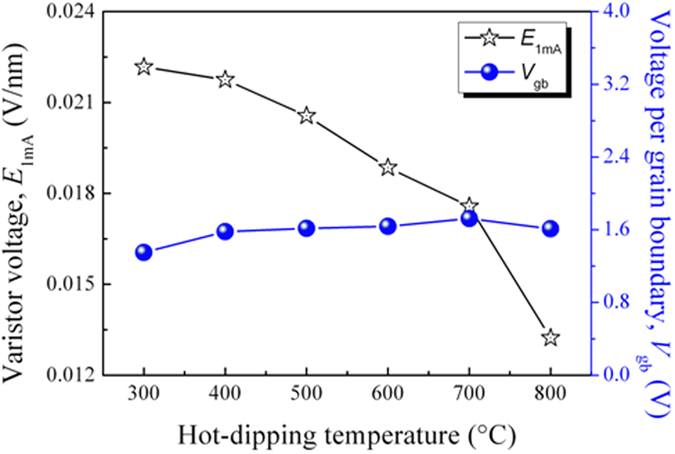
Varistor voltage and voltage per grain boundary of the zinc oxide film varistors as a function of hot-dipping temperature.

The varistor voltage (*E*_1mA_) and voltage per grain boundary (*V*_gb_) of the zinc oxide film varistors as a function of hot-dipping temperature are illustrated in Fig. 8. The varistor voltage sharply decreased monotonously from 0.02217 to 0.01623 V/nm with the increase of hot-dipping temperature (black line marked with stars). It is well-known that the breakdown voltage (varistor voltage) of zinc oxide varistors depends on the size of zinc oxide grains in the samples and the applied voltage per grain boundary^26^. But from Fig. 8, it can be seen that, with increasing hot-dipping temperature, the applied voltage per grain boundary changed in a very narrow range (1.35–1.72, see the blue line marked with balls). And the varistor voltage of the present zinc oxide film varistors is in inverse proportion with the size of the zinc oxide grains in the films (see Fig. 2h). Thus, the sudden drop of varistor voltage with increasing hot-dipping temperature is attributed to the decrease in the number of effective grain boundaries caused by the increase in the ZnO grain size.

In summary, when the samples were hot-dipped in Pr_6_O_11_ at 700 °C for 50 min in air, the prepared zinc oxide thin-film varistor presents the maximum nonlinear coefficient of 39.29, the minimum leakage current of 0.02736 mA/cm^2^, and a varistor voltage of 0.01757 V/nm.

## Electrical conducting mechanism

**Figure 9:**
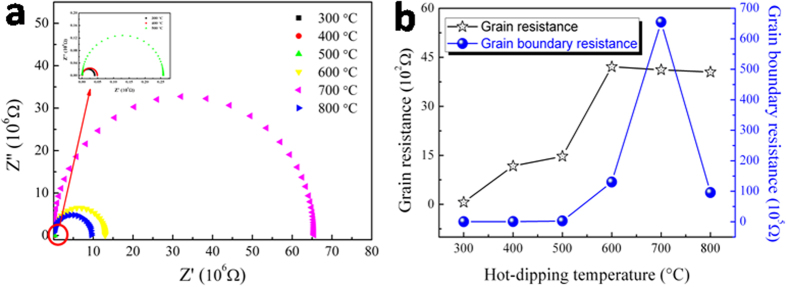
(**a**) Complex impedance spectra, and (**b**) grain and grain boundary resistances of the zinc oxide films samples hot-dipped in Pr_6_O_11_ for 50 min in air at different temperatures.

Impedance spectroscopy was used to characterize the different microstructure regions of the zinc oxide films samples prepared by hot-dipping in Pr_6_O_11_ for 50 min in air at different temperatures. Typical complex impedance spectra for the present varistor materials, Z” versus Z’, are displayed in Fig. 9a. From this figure, the grain and grain boundary resistances of the zinc oxide film samples hot-dipped at different temperatures are shown in Fig. 9b. As is seen from this figure, with the increase in hot-dipping temperature, the grain resistance of the samples gradually increased, reaching a plateau when the temperature was higher than 600 °C (black line marked with stars), while the grain boundary resistance initially increased and then decreased, achieving a maximum when the temperature was 700 °C (blue line marked with balls).

As discussed in Sections 3.2 and 3.3, microstructurally, the hot-dipping of oxygen-deficient zinc oxide thin films in Pr_6_O_11_ will give rise to a structure consisting of semiconducting zinc oxide grains surrounded by very thin insulating intergranular Pr_6_O_11_ layers. Accordingly, as shown in Fig. 9a, the negative impedance diagram describes the grain resistance and grain boundary resistance. With the increase in the hot-dipping temperature, the oxidation of oxygen-deficient zinc oxide films would become more and more completed, which would result in less oxygen vacancies in the films and thus increase the grain resistance (see Fig. 9b). Because of the existence of oxygen vacancies in the oxygen-deficient zinc oxide films, under the action of applied electric field, the adjacent electrons will fill the vacancies, leaving new spaces in the original positions of electrons. After that, other electrons would transfer to the newly created spaces. As a result of the whole process, a certain amount of charges were transferred (conducting). This process is called vacancy conduction. Since the number of defects (oxygen vacancies) decreased with the increase of hot-dipping temperature, electrons would be difficultly transferred via vacancy, thus reducing the charges transferred. Consequently, the electrical resistivity of the zinc oxide films would increase with increasing hot-dipping temperature. As for the plateau of grain resistance when the temperature was higher than 600 °C, it is because the samples were gradually oxidized completely with a composition of zinc oxide close to the stoichiometric ZnO, which possesses the highest grain resistance in all forms of oxygen-deficient zinc oxides.

With regards to the change of grain boundary resistance with the hot-dipping temperature (see the blue line marked with balls in Fig. 9b), it can be explained as follows. When it was lower than 700 °C, the grain boundary resistance increased gradually with increasing hot-dipping temperature. This is because Pr_6_O_11_ permeated into the boundary of the zinc oxide grains in the film varistor samples during the hot-dipping, forming an insulating layer at the grain boundary, and with increasing hot-dipping temperature, the insulating layer became more and more completed, thus resulting in increased grain boundary resistance. But if it was higher than 700 °C, with further increased hot-dipping temperature, the grain boundary resistance of the samples decreased gradually. This phenomenon is correlated with the separation of Pr_6_O_11_ from the grain boundaries in the varistors at enhanced temperatures, which will destroy the completeness of the intergranular insulating Pr_6_O_11_ layer, thus decreasing the electrical resistance of the grain boundary. When the film was hot-dipped in Pr_6_O_11_ at 700 °C for 50 min in air, the resultant sample would present a maximum value of grain boundary resistance, the maximum contribution of grain boundary resistance to the total resistance, because in such case an intergranular insulating layer with perfect structure was formed. Because the nonlinearity of a varistor is strongly dependent on the resistivity of the grains and grain boundary, low value of grain resistance and high value of grain boundary resistance will lead to a high nonlinear coefficient^27^. Therefore, the sample prepared by hot-dipping ZnO_0.81_ thin films in Pr_6_O_11_ at 700 °C for 50 min in air presented the optimum nonlinear performance, although its grain resistance is relatively large.

## Varistor stability

**Figure 10:**
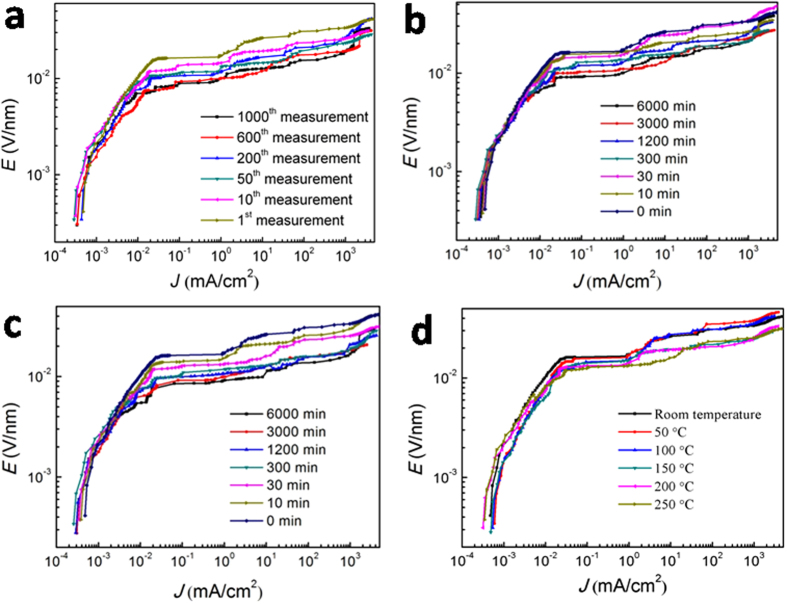
*E-J* characteristic curves of the zinc oxide film varistors during stability tests. (**a**) when it was measured at room temperature for 1000 times of repeating charge-discharge cycles; **(b,c),** when it was applied at 100 and 250 °C for up to 100 h, respectively; and (**d**) when it was applied at a temperature up to 250 °C, during which at each temperature, the sample was soaked at least for 5 min before measurement. All the samples were prepared under the optimum conditions of hot-dipping ZnO_0.81_ thin films in Pr_6_O_11_ at 700 °C for 50 min in air.

There are many factors affecting the stability of ZnO varistors. When a varistor is working, it would always bear a certain steady-state voltage. Thus, as a resistor, the number of charging-discharging cycle and working temperature it suffers from often present a significant impact on its nonlinear characteristics. In order to investigate the varistor stability for the present ZnO-Pr_6_O_11_ thin film samples, with the samples prepared under the optimum condition (by hot-dipping ZnO_0.81_ thin films in Pr_6_O_11_ in air at 700 °C for 50 min), their *E-J* curves were recorded after the samples were charged-discharged at room temperature for 1000 times, heated at 100 or 250 °C for up to 100 h, and applied at up to 250 °C, respectively. The results are presented in Fig. 10.

Figure 10a represents the *E-J* characteristic curves of a typical sample measured during repeating charge-discharge cycles. It is seen from this figure that the nonlinearity of the present ZnO-Pr_6_O_11_ thin-film varistor is recoverable after charge-discharge for 1000 times, indicating that the samples have good stability for repeating charge-discharge. Figure 10b and c displays the *E-J* plot of the samples at an operation temperature of 100 and 250 °C for up to 100 h, respectively. This plot indicates that at enhanced application temperature up to 250 °C, the nonlinearity of the present ZnO-Pr_6_O_11_ thin-film varistor is steady even after applying for 100 h, revealing that the samples have good stability for application at enhanced temperature. And Fig. 10d exhibits the *E-J* characteristic curves of a typical sample measured at varying temperature. It can be seen that the increase of application temperature up to 250 °C almost presents no effect on the nonlinearity of the present ZnO-Pr_6_O_11_ thin-film varistor, indicating that the samples have good stability for application at varying temperature.

When a ZnO varistor is working, it should withstand a certain steady-state voltage, because the heat produced when a current passes through a resistor can’t dissipate easily, and the temperature of the varistor will rise rapidly. After maintaining the initial steady state, traditional ZnO varistors quickly reached the possible heat breakdown conditions, the so-called aging. Therefore, the stability of a ZnO varistor at high-temperature operation is very important. In the present work, after charge-discharge at room temperature for 1000 times, heating at 100 or 250 °C for up to 100 h, or applying at up to 250 °C, the varistors still performed well. Therefore, such nanoscaled thin-film varistors will be very promising in electrical/electronic devices working at low voltage.

In summary, high-performance ZnO-Pr_6_O_11_ thin-film varistors were fabricated simply by hot-dipping oxygen-deficient zinc oxide thin films in Pr_6_O_11_ powder. The applied zinc oxide films had a composition of ZnO_0.81_ and a thickness of about 200 nm, which were deposited onto conducting silicon chips by radio frequency magnetron sputtering of a sintered zinc oxide ceramic target. Then the films were hot-dipped in Pr_6_O_11_ at a temperature from 300 to 800 °C for an optimized time of 50 min in air. With the increase of hot-dipping temperature, the varistor voltage of the samples decreased from 0.02217 to 0.01623 V/nm, owing to the decreased number of effective grain boundaries in the varistors. The nonlinear coefficient increased initially and then decreased, presenting a recorded maximum of 39.29, when the temperature was 700 °C; correspondingly, the leakage current density first decreased and then increased, reaching a minimum of 0.02736 mA/cm^2^, owing mainly to the formation and destroying of complete zinc oxide/Pr_6_O_11_ grain boundaries. And after charge-discharge at room temperature for 1000 times, heating at 100 and 250 °C for up to 100 h, or applying at up to 250 °C, the varistor performance of the samples was still well kept. Due to its low sensitive voltage, high nonlinear coefficient, low leakage current, and high stability, the present nanoscaled thin-film varistors would be very promising in electronic/electrical devices working at low voltage.

## Methods

### Samples preparation

The applied zinc oxide films had a composition of ZnO_0.81_ and a thickness of about 200 nm, which were deposited onto conducting silicon chips by reactive RF magnetron sputtering of a sintered zinc oxide ceramic target at ambient temperature. For details, please check them in refs 6 and 11. Then the as-deposited ZnO_0.81_ thin film samples were buried in Pr_6_O_11_ powder in a half-covered alumina crucible, and heated in a muffle furnace at a temperature of 300, 400, 500, 600, 700 and 800 °C, respectively, for an optimized time of 50 min (see Extended Data Fig. 3). After the hot-dipping, the samples were cooled down naturally to room temperature simply by shutting down the electricity of the furnace. Finally, silver paste was daubed at room temperature on the chip surface as electrodes for the measurement of room-temperature electrical properties. But for the varistor stability tests, the silver paste was toasted at 500 °C onto the samples in order to avoid the diffusion of the electrode materials during the measurement.

### Materials characterization

The elemental composition and chemical state of the samples were measured by a Thermo Fisher X-ray photoelectron spectroscope (non-monochromated Al Kα radiation, photon energy 1486.7 eV). And the spectrometer was calibrated by the binding energy of C1s line (284.6 eV). The microstructure of the samples was examined on their surfaces and fractural cross-sections by FE-SEM (LEO-1530). From the obtained SEM images, the grain size and film thickness of the samples were evaluated by the Nano Measurer software. The elemental distribution in the samples was analyzed by an attached EDX system. The phase composition of the samples was identified by grazing incidence XRD (GI-XRD, D/max-RB, Cu Kα radiation, and λ = 1.5418 Å) through a continuous scanning mode at a speed of 6°/min with an X-ray incidence angle of 0.5°.

### Measurements of electrical properties

The *E-J* characteristics of the samples were recorded by a Keithley 2410 Multimeter. Through the recorded *E-J* curves, the nonlinear coefficient (*α*) of the samples was calculated according to the following equation:


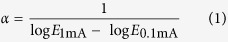


where the *E*_1mA_ and *E*_0.1mA_ are the electric fields corresponding to the current densities of 1.0 and 0.1 mA/cm^2^, respectively. The electric field at the current density of 1.0 mA/cm^2^ was defined as the varistor voltage (*E*_1mA_), and the leakage current (*I*_L_) was determined at 0.75·*E*_1mA_. Excepting the varistor stability tests, all the *E-J* curves were measured at room temperature with each sample for one-time testing. But for the varistor stability tests of the optimum samples, their *E-J* curves were recorded after the samples were charged-discharged at room temperature for 1000 times, heated at 100 and 250 °C for up to 100 h, and applied at up to 250 °C, respectively. The applied voltage per grain boundary (*V*_gb_) was calculated using,


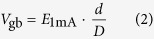


where *E*_1mA_ is the varistor voltage of the prepared film varistors, *d* is the average size of zinc oxide grains, and *D* is the thickness of the varistor thin films. The complex impedance spectra of the samples were recorded with an electrochemical workstation (CHI660E, CH Instrument Company, China) under the frequency ranging from 1 to 10^5^ Hz at an amplitude voltage of 20 mV. The impedance data were analyzed with the ZSimpWin program.

## Additional Information

**How to cite this article**: Wang, Y. *et al*. High-performance varistors simply by hot-dipping zinc oxide thin films in Pr_6_O_11_: Influence of temperature. *Sci. Rep.* **7**, 41994; doi: 10.1038/srep41994 (2017).

**Publisher's note:** Springer Nature remains neutral with regard to jurisdictional claims in published maps and institutional affiliations.

## Supplementary Material

Supplementary Information
