# Supplementary material for: High-performance varistors simply by hot-dipping zinc oxide thin films in Pr6O11: Influence of temperature
Source: Sci Rep. 2017 Feb 3;7:41994. doi: 10.1038/srep41994 (PMC5290466; doi:10.1038/srep41994)
Supplement: Supplementary Information [file srep41994-s1.pdf]

SUPPLEMENTARY INFORMATION

High-performance varistors simply by hot-dipping zinc oxide thin films in Pr_6O_{11}: Influence of temperature

Yang Wang[1,2], Zhijian Peng[1*], Qi Wang[1,2], Chengbiao Wang[1], Xiuli Fu[2*]

[1]School of Engineering and Technology, China University of Geosciences, Beijing 100083, PR China. Tel: 86-10-82320255; Fax: 86-10-82322624; E-mail: pengzhijian@cugb.edu.cn (Z.J.P.)

[2]State Key Laboratory of Information Photonics and Optical Communications, and School of Science, Beijing University of Posts and Telecommunications, Beijing 100876, P. R. China. Tel: 86-10-62282452; Fax: 86-10-62282054; E-mail: xiulifu@bupt.edu.cn (X.L.F.)

We would like to thank the financial support for this work from the National Natural Science Foundation of China (grant nos. 61274015, 11674035, and 11274052), Excellent Adviser Foundation in China University of Geosciences from the Fundamental Research Funds for the Central Universities, and Fund of State Key Laboratory of Information Photonics and Optical Communications (Beijing University of Posts and Telecommunications).

Yang Wang, et al, Extended Data Figure 1

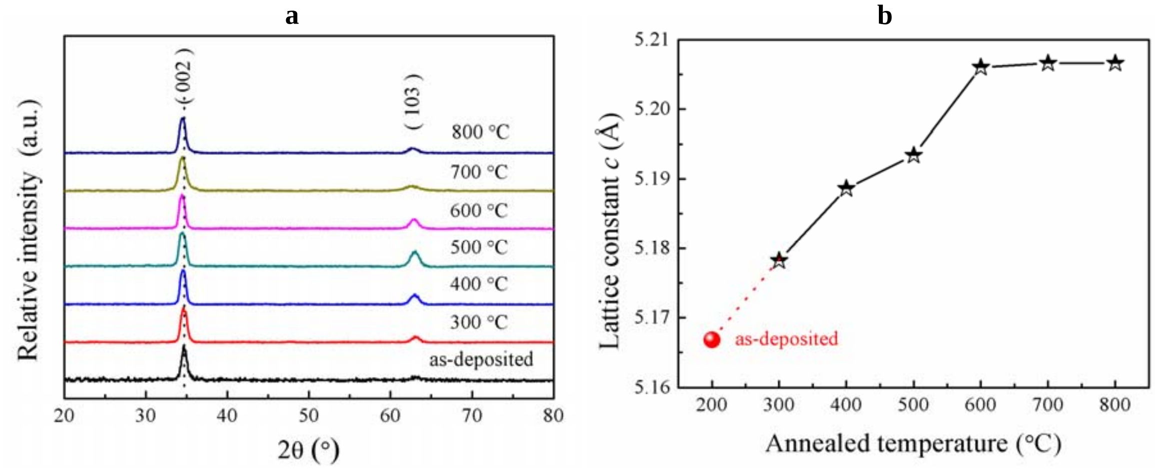

Extended Data Figure 1 | XRD results for the annealed ZnO_{1-x} film samples. **a,** XRD patterns for the annealed samples prepared at the same temperature as those of the hot-dipped samples in air for 50 min. **b,** Lattice constant *c* calculated from the recorded XRD patterns. For comparison, the result on the as-deposited ZnO_{1-x} film is also presented. It can be seen that, when the hot-dipping temperature increased from 300-800 °C, the lattice constant increased more and more slowly, approaching to 5.2066 Å (the value of stoichiometric ZnO), implying that the samples were gradually oxidized completely with a composition of zinc oxide close to the stoichiometric ZnO.

Yang Wang, et al., Extended Data Figure 2

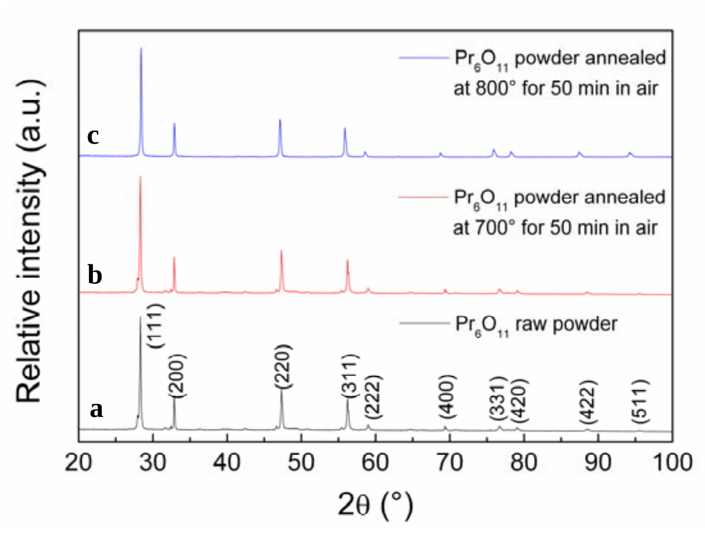

Extended Data Figure 1 | XRD patterns for praseodymium oxide powders. **a,** XRD pattern for the applied raw Pr_6O_{11} powder in this work, **b,** XRD pattern for the Pr_6O_{11} powder annealed at 700 °C for 50 min in air, and **c**, XRD pattern for the Pr_6O_{11} powder annealed at 800 °C for 50 min in air. All the diffraction peaks of three samples can be assigned to the same Pr_6O_{11} phase (JCPDS card no. 42-1121), indicating that Pr_6O_{11} would be stable during the present annealing at up to 800 °C for 50 min in air.

Yang Wang, et al., Extended Data Figure 3

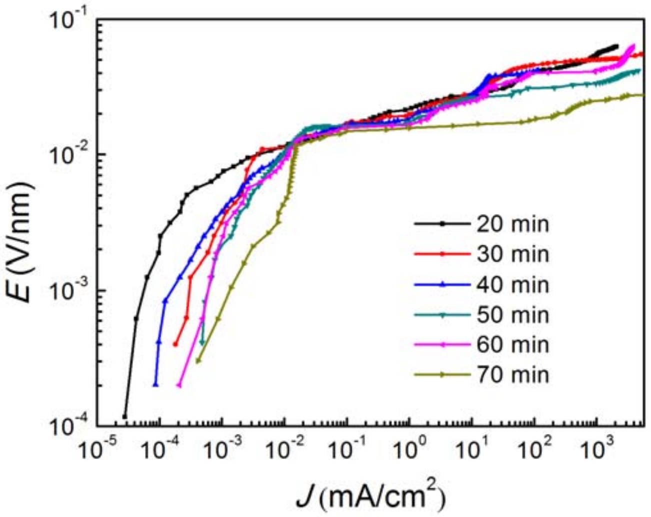

Extended Data Figure 3 | Influence of hot-dipping times. The *E-J* characteristic curves were recorded on the varistors prepared by hot-dipping the as-deposited zinc oxide films in Pr_6O_{11} at 700 °C for different times. From this figure, the *E-J* characteristic parameters (including the nonlinear coefficient α, leakage current I_L, and varistor voltage E_{1mA}) of the varistors can be calculated, and the results are listed in the following table. It can be seen from this table, after hot-dipping in Pr_6O_{11} at 700 °C for 50 min, the optimum varistor performance can almost be reached.

Time (min)	α	I_L (mA/cm^2)	E_{1mA} (V/nm)
20	8.85	0.09435	0.02196
30	13.54	0.04269	0.01982
40	22.47	0.02202	0.01807
50	39.23	0.01556	0.01723
60	39.14	0.01582	0.01663
70	39.59	0.01516	0.01575
